# Living in isolation – population structure, reproduction, and genetic variation of the endangered plant species *Dianthus gratianopolitanus* (Cheddar pink)

**DOI:** 10.1002/ece3.1611

**Published:** 2015-08-07

**Authors:** Christina M Putz, Christoph Schmid, Christoph Reisch

**Affiliations:** Institute of Botany, University of Regensburg93040, Regensburg, Germany

**Keywords:** Amplified fragment length polymorphisms, Dianthus gratianopolitanus, distribution range, genetic variation, isolation, population structure, rarity, reproductive traits

## Abstract

The endangered plant species *Dianthus gratianopolitanus* exhibits a highly fragmented distribution range comprising many isolated populations. Based upon this pattern of distribution, we selected a study region in Switzerland with a lower magnitude of isolation (Swiss Jura) and another study region in Germany with a higher degree of isolation (Franconian Jura). In each region, we chose ten populations to analyze population structure, reproduction, and genetic variation in a comparative approach. Therefore, we determined population density, cushion size, and cushion density to analyze population structure, investigated reproductive traits, including number of flowers, capsules, and germination rate, and analyzed amplified fragment length polymorphisms to study genetic variation. Population and cushion density were credibly higher in German than in Swiss populations, whereas reproductive traits and genetic variation within populations were similar in both study regions. However, genetic variation among populations and isolation by distance were stronger in Germany than in Switzerland. Generally, cushion size and density as well as flower and capsule production increased with population size and density, whereas genetic variation decreased with population density. In contrast to our assumptions, we observed denser populations and cushions in the region with the higher magnitude of isolation, whereas reproductive traits and genetic variation within populations were comparable in both regions. This corroborates the assumption that stronger isolation must not necessarily result in the loss of fitness and genetic variation. Furthermore, it supports our conclusion that the protection of strongly isolated populations contributes essentially to the conservation of a species' full evolutionary potential.

## Introduction

Rare plant species are largely subjected to the effects of isolation (Silcock and Fensham 2014). The impact of isolation on so-called new rare species (Huenneke 1991), which have been formerly widespread and are now rare, as a result of landscape fragmentation processes, has been analyzed in numerous studies during the past decades (Hooftman et al. 2004; Galeuchet et al. 2005; Schleuning et al. 2009). It is widely accepted that the fragmentation of formerly common species due to anthropogenic land-use changes results in stronger geographic isolation of smaller populations (Ouborg et al. 2006). As a result, the exchange of pollen and seeds between populations is restricted, which means that gene flow is limited (Listl and Reisch 2014). Therefore, genetic variation within populations decreases, while genetic variation between populations increases. In previous studies, it has been shown that population size, fitness, and genetic variation are strongly correlated (Leimu et al. 2006). The loss of genetic variation may, therefore, lead to impaired generative (Schmidt and Jensen 2000) or diminished vegetative performance (de Jong and Klinkhamer 1994; Chaloupecká and Lepš 2004). This potentially causes an increased susceptibility to pathogens and herbivores on the short term (Ellstrand and Elam 1993; Schmid and Matthies 1994), and a diminished evolutionary capacity to environmental changes, increasing extinction probability on the long term (Matthies et al. 2004; Ouborg et al. 2006).

However, naturally fragmented and isolated populations of “old rare species” (Huenneke 1991), which have been always rare and occur in special habitats, have much less been in the research focus during the last decades (Hensen et al. 2010; Vogler and Reisch 2013). Previously, it has been demonstrated that rare species are less susceptible to the effects of fragmentation than common species (Honnay and Jacquemyn 2007). This is supported by the results of a study about isolated populations of alpine plant species (Kuss et al. 2008). The authors of this study originally assumed that genetic variation should decrease with increasing isolation. However, they detected high levels of genetic variation also in strongly isolated populations of alpine plant species. The authors therefore concluded “that natural fragmentation does not necessarily result in particularly high levels of mean genetic population differentiation or in a loss of genetic diversity within populations” (Kuss et al. 2008).

The population structure and reproduction of naturally rare species has less been investigated than genetic variation (García et al. 2000; Dorken and Eckert 2001; Jump and Woodward 2003). Most of these studies analyzed population structure and reproduction in the context of the abundant center model (ACM), which postulates that populations of a species become progressively smaller, less dense, less frequent, and more spatially isolated approaching the edge of a species' range (Lawton 1993; Sagarin and Gaines 2002; Sagarin et al. 2006). It is assumed that, as a result of this pattern, levels of reproduction and recruitment decrease among isolated populations. This assumption is supported by several studies of “new rare species”, which showed that, for example, pollination success decreases with increasing isolation and that fragmentation in general affects the reproductive success (Jennersten 1988; Kolb 2005).

*Dianthus gratianopolitanus* occurs on naturally isolated limestone outcrops (Hegi 1986) and exhibits a highly fragmented distribution range comprising many strongly isolated populations (Meusel et al. 1978; Käsermann 1999). The populations of the species occur continuously throughout the center of the distribution range in the Swiss Jura but become more and more isolated toward the periphery of the distribution range. The most peripheral populations are hundreds of kilometers distant to other populations (Käsermann 1999). Based upon this pattern of distribution, we selected two study regions from the distribution range of the species with a different magnitude of isolation to analyze population structure, reproduction, and genetic variation in a comparative approach.

Based upon the outcome of previous studies about the effects of isolation on recently fragmented populations of formerly widespread species (Fahrig 2003; Jacquemyn et al. 2007; Schleuning et al. 2009), we expected that increasing isolation should reduce population size and density and the reproduction of *D. gratianopolitanus*. Moreover, genetic variation within populations should be decreased, while genetic variation between populations should be increased.

## Methods

### Study species

*Dianthus gratianopolitanus* Vill. (Hegi 1986) is a perennial, long-lived plant which forms cushions or mats consisting of numerous hemi-rosettes (Käsermann 1999). The species colonizes rocky ridges and outcrops mainly of limestone (Hegi 1986). Besides vegetative reproduction by shoots, conspicuous pale to dark pink and odorant flowers attract diurnal butterflies as well as diurnal and nocturnal moths between end of May and June (Erhardt 1990). Each pollinated flower generates a capsule containing anemochorous diaspors (Käsermann 1999).

*D*. *gratianopolitanus* is a fairly rare pre-alpine plant endemic to central Europe with a highly fragmented distribution, which seems to be relic from periods previous to the last glaciations (Erhardt 1990). The current distribution center is a continuous area along the Jura Mountains (French, Swiss, and Swabian Jura), but the species also exhibits more isolated populations in low mountain ranges of middle and eastern France, southern Belgium, Germany, Bohemia, and the west-polish lowland. The most isolated population is found in south England (Cheddar Gorge, N. Somerset) (Käsermann 1999).

Although *D*. *gratianopolitanus* is naturally rare, more and more populations decline and disappear, mainly due to the effects of hiking and climbing (Welk 2002), because they are overgrown by shrubs and trees and may be also due to climatic changes and the input of nitrogen. The species is therefore listed in the German red data book with category three (Korneck et al. 1996).

### Study design

**Figure 1:**
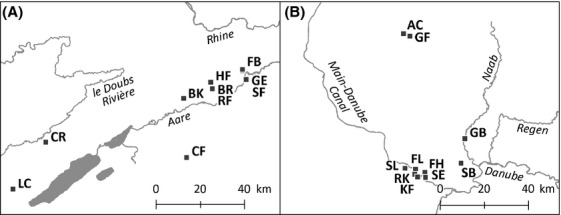
Geographic position of the investigated populations in the two study regions in Switzerland (A) and Germany (B).

We selected for our investigation two study regions, where the populations of *D*. *gratianopolitanus* exhibited a different magnitude of isolation. One region was located in Switzerland (Swiss Jura), near to the potential center of the distribution range, and the other study region was located in Germany (Franconian Jura), more closely to the periphery of the distribution range. From each region, we analyzed each ten populations (Fig.1).

The magnitude of isolation was characterized based upon the quadrants of the grid of topographic maps (TK 25, scale 1:25000), which is officially used for the design of species distribution maps in Germany. This grid was extended to Switzerland and the locations, where the species occurs in Switzerland were transferred from the Swiss distribution map in the accordant quadrants. Based upon this dataset, we determined the number of quadrants within a radius of 30 km around the selected study populations, where *D*. *gratianopolitanus* occurs. The two study regions differed significantly in the magnitude of isolation (*U* test: *P* = 0.000). In Switzerland, *D*. *gratianopolitanus* occurred on average in 33.5 quadrants around the selected populations, whereas in Germany, the species was recorded on average in 12.2 quadrants around the study populations. The magnitude of isolation was, therefore, higher in Germany than in Switzerland.

In both regions, we studied 15 individuals (cushions) per population, which were randomly chosen across the colonized habitat and represents about 50% of the number of individuals per population. However, in the smallest population, only 12 cushions were available (Table1). All data were collected within one vegetation period from May to July.

**Table 1:** Analyzed populations of *D. gratianopolitanus* with their number, label, study region (S, Switzerland; G, Germany), number of sampled individuals (*n*), and altitude (m a. s. l.). For conservation reasons, we refrain from presenting the exact geographic coordinates (latitude and longitude). Data are, however, available from the authors

No.	Label	Population	Area	n	Altitude
Switzerland					
1	LC	Le Chasseron	S	15	1600
2	CR	Col de Roches	S	15	910
3	CF	Chrüzflue	S	15	700
4	BK	Balmfluechöpfli	S	15	1250
5	RF	Ravellen	S	15	620
6	BR	Bränten	S	15	600
7	HF	Holzflue	S	15	730
8	GE	Geissflue,	S	15	600
9	FB	Ruine Froburg	S	15	830
10	SF	Säliflue	S	15	530
Germany					
11	SL	Schwedenleite	G	15	480
12	FL	Fischleiten	G	15	470
13	RK	Rosskopf	G	15	460
14	KF	Kachelfelsen	G	15	450
15	FH	Falkenhorst	G	15	450
16	SE	Schleuse	G	15	450
17	SB	Steinerbrückl	G	12	400
18	GB	Grain am Berg	G	15	350
19	AC	Achtel	G	15	420
20	GF	Grundfelsen	G	15	490

### Population structure

The number of individuals in each population was enumerated by inspecting the whole habitat area, thereby one spatially separated cushion was regarded as one individual. The size of the habitat area (e.g., rocky ridge) was estimated using topographic maps. Based upon the size of the area and the number of individuals, population density was calculated as cushions per square meter and is, therefore, an indicator for habitat occupancy. Furthermore, width and length of each individual was measured to calculate the cushion size in cm². Cushion density was estimated as the number of shoots per square meter, counted in small plots of 10 × 10 cm and extrapolated to 1 m².

### Reproductive traits

In the field, the number of flowers and capsules of each investigated cushion were counted in May and July, respectively. Using these data, we calculated fruit set, as number of capsules divided by number of flowers. Seeds were collected from the selected individuals with regard to nature conservation, which means that we did not collect all available seeds, but only 20% of the seeds to allow natural reproduction in the population. As populations of the species are generally small, we obtained not enough seeds to test all germination treatments for all populations separately. Therefore, tests were conducted with pooled seeds from the two study regions, respectively, to receive a sufficient number of replications. Different treatments were used to evaluate the germination response of seeds from Swiss and German populations associated with different environmental conditions which indicate a gap detection mechanism and longevity of the seeds. In this way, we tested whether differences between the two study regions occur at different treatments. We analyzed germination at four treatments, which were generally used by default in germination ecological analyses, and have turned out to be the most suitable conditions to test the germination of central European plants. They are derived from typical temperatures at day and night (Baskin et al. 2006). We applied three treatments with an 8 h light period: constant 14°C, constant 22°C, and fluctuating 22°C day/14°C night. A further germination test was performed under 22°/14°C fluctuating conditions in darkness.

Seeds were placed uniformly in petri dishes (92 × 16 mm) lined with a double layer of filter paper (Sartorius, Ø90 mm) moistened with 5 mL of distilled water. To prevent evaporation, each petri dish was sealed separately with parafilm. Per treatment eight petri dishes as replicates with 10 seeds each were kept in stacks of eight and rotated daily. All experiments were placed in an incubator (Licht-Thermostat, Rubarth Apparate GmbH) with a constant humidity of 65%. For germination in darkness, the petri dishes were kept in a black box and scored under green light (25 W) to exclude any influence of light of a different wavelength. Germination was recorded daily for the first 10 days, then twice a week for a total of 6 weeks. Germinated seeds were removed.

### Genetic variation

Shoots with young, green leaves were sampled of 15 cushions per populations for genetic analysis, and in total, 297 individuals were analyzed. Single shoots were placed in permeable bags, immediately stored in sealed containers and dried on silica gel. Genomic DNA was isolated from dry leaf material using the CTAB-based method (Rogers and Bendich 1994) as described before (Reisch and Kellermeier 2007). Concentrations of the DNA extracts were measured photometrically. DNA solutions were diluted with water for molecular biology to 7.8 ng/*μ*L and used for the analysis of amplified fragment length polymorphisms (AFLPs), which were conducted concordant with the protocol from Beckmann Coulter as described previously (Bylebyl et al. 2008; Reisch 2008). Three primer combinations were chosen for a subsequent selective PCR. For detection, EcoRI primers labeled with different fluorescent dyes (M-CAT/D2-E-ACC, M-CAT/D3-E-ACG, M-CTT/D4-E-ACT; Beckman Coulter, Krefeld, Germany) were used. Selective PCR products were diluted threefold (D2), twofold (D3), and eightfold (D4) with 1× TE_0.1_ buffer for AFLP. After redissolving the pelleted DNA in a mixture of 24.8 *μ*L sample loading solution (SLS, Beckman Coulter) and 0.2 *μ*L CEQ Size Standard 400 (Beckman Coulter), selective PCR products were separated by capillary gel electrophoresis on an automated sequencer (CEQ 8000, Beckmann Coulter).

Results were examined using the CEQ 8000 software (Beckman Coulter) and analyzed using the software Bionumerics 6.6 (Applied Maths, Kortrijk, Belgium). From the computed gels, only those fragments were taken into account that showed intense and articulate bands. Samples yielding no clear banding pattern or obviously representing PCR artefacts were repeated or finally excluded. Reproducibility of molecular analyses was investigated with 10% of all analyzed samples by means of estimating the genotyping error rate (Bonin et al. 2004), which was 3.2%.

### Statistical analysis

From the AFLP bands, a binary (0/1) matrix was created wherein the presence of a fragment of a given length was counted as 1 and the absence as 0. The final matrix and all further calculations comprised 297 samples. Employing the software PopGene 1.32 (Yeh and Layton 1979), genetic variation within populations was computed as the percentage of polymorphic loci %PL, as Nei's gene diversity H_e_ (H = 1– sum(p_i_)^2^) and as Shannon's Information Index SI (SI = sum(p_i_)ln(p_i_); p_i_ = allele frequency). The apportionment of genetic variation within and between populations and subpopulations was assessed by hierarchical AMOVA with the software GenAlEx 6.3 (Peakall and Smouse 2001). In a Mantel test (999 permutations), the matrix of pairwise genetic distances (Φ_PT_) was correlated with a matrix of the respective geographic distances (km) among populations (Mantel 1967).

Population parameters were compared between regions by means of Bayesian two-group models. The analysis was carried out using Markov Chain Monte Carlo sampling (MCMC) with the JAGS 3.4.0 software package (Plummer 2003). Habitat area was modeled as being gamma-distributed, and all other parameters could be approximated by Student's *t*-distributions. Population density and the number of capsules had to be square root transformed prior to analyses. All models were executed with four chains and afterward checked for chain convergence. Highest density intervals (HDI) were computed for the group mean difference and considered credible, when the HDI excluded zero. All estimated parameters had an effective sample size (ESS) of >10 k.

For the analysis of the seed germination experiment, a Bayesian approach was chosen for both its higher flexibility and informative value as compared to classical NHST procedures. Only data of day 7 were analyzed, when overall germination was close to 50%. Modeling and interpretation were carried out using the software packages R 3.2.0 (R-Core-Team, 2013) and RStan 2.6.0 for Hamiltonian Monte Carlo (HMC) sampling (Stan-Development-Team, 2014) as well as utility functions provided by Kruschke (2015). The model involved a hierarchical two-way design with the logistic as the inverse link function. Besides the deflection parameters and their corresponding standard deviations, the model estimated the germination per factor combination as being beta distributed with equal concentration parameters between cells. This is a Bayesian analogue to a two-way logistic ANOVA. The complete and commented Stan model specification is available from the supplementary material. All priors were set to be uninformed using vague gamma and normal distributions. Sampling was carried out with four HMC chains with 500 k steps each with thinning set to every 25th step, a burn-in period of 1000 steps and 500 steps for adaption. All parameters were checked for chain convergence. Autocorrelation was assessed as the ESS aiming at a lower limit of 10 k for the relevant parameters. Differences in germination between treatments and regions, respectively, were subsequently analyzed using contrasts of the corresponding marginal distributions.

We tested the relationship between population structure parameters, reproductive traits, and genetic variation using correlation analyses based on Spearman's rank correlation coefficient with IBM SPSS Statistics 20 (IBM Corp.) for Windows.

## Results

### Population structure

**Figure 2:**
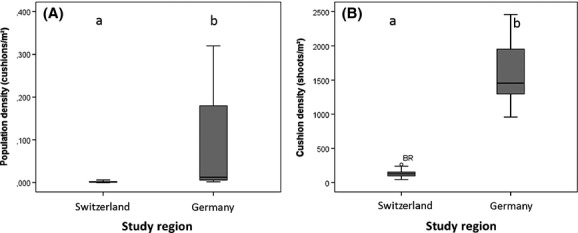
Population density and cushion density of *D. gratianopolitanus* of populations from Switzerland and Germany with significant differences: (A) population density and (B) cushion density.

The analyzed populations of *D. gratianopolitanus* occurred in credibly smaller habitat areas in Switzerland than in Germany. However, the number of individuals (12–60) was quite consistent in both regions (Table2). Hence, mean population density differed credibly (Fig.2A) between Swiss and German populations (0.002 vs. 0.078 cushions per m²). In Switzerland, 0.0063–0.0001 cushions occurred per square meter compared to a variation between 0.0050 and 0.3200 cushions in Germany.

**Table 2:** Occurrence of *D. gratianopolitanus* within a 30 km radius, habitat area, and population structure of all investigated populations. Mean values for the study regions and results of the statistical analysis are given. (NQ, number of quadrants of the topographic maps, where the species occurs within a 30 km radius around the studied populations; HA, habitat area in m²; NI, number of individuals; PD, population density in cushions per m²; CS, cushion size in cm²; CD, cushion density in shoots per m²)

No.	Abbr.	Population	HA	NI	PD	CS	CD
Switzerland							
1	LC	Le Chasseron	49,600	31	0.0006	1455	96
2	CR	Col de Roches	12,100	15	0.0012	181	155
3	CF	Chrüzflue	3800	24	0.0063	2013	43
4	BK	Balmfluechöpfli	12,2100	18	0.0001	1526	80
5	RF	Ravellen	20,300	27	0.0013	301	238
6	BR	Bränten	7900	16	0.0020	718	263
7	HF	Holzflue	95,800	31	0.0003	1445	116
8	GE	Geissflue	21,600	16	0.0007	510	138
9	FB	Ruine Froburg	6600	29	0.0044	1448	146
10	SF	Säliflue	6300	21	0.0033	952	113
		Mean	34,610	23	0.002	1055	139
Germany							
11	SL	Schwedenleite	9200	20	0.0022	518	1446
12	FL	Fischleiten	4400	60	0.0136	1932	2457
13	RK	Rosskopf	1900	23	0.0121	1783	1297
14	KF	Kachelfels	75	16	0.2133	857	957
15	FH	Falkenhorst	5800	61	0.0105	1522	1307
16	SE	Schleuse	7000	43	0.0061	2017	2306
17	SB	Steinerbrückl	2400	12	0.0050	658	1465
18	GB	Grain am Berg	1900	23	0.0121	1385	1952
19	AC	Achtel	50	16	0.3200	1770	1560
20	GF	Grundfelsen	100	18	0.1800	1905	1246
		Mean	3283	29	0.078	1435	1599
Most credible difference:HDI			**13,664 [5367, 22223]**	5.63 [−9.51, 20.9]	**0.025 [0, 0.097]**	406 [−252, 1030]	**1460 [1070, 1830]**
Effect size *r*			**0.56**	0.22	**0.53**	0.35	**0.91**

Most credible differences are given in bold.

Mean cushion size did not differ credibly between Switzerland and Germany although cushions were smaller in Switzerland (1055 cm²) than in Germany (1435 cm²). Generally the size of the cushions varied between 181 and 2017 cm². However, mean cushion density (Fig.2B) of individuals from Switzerland (139 shoots per m²) was credibly lower than mean cushion density of individuals from Germany (1599 shoots per m²). In Swiss populations, a density of maximal 263 shoots per m² was evaluated, which is in sharp contrast to the German populations consisting of at least 957 shoots per m² (Table2).

### Reproductive traits

**Figure 3:**
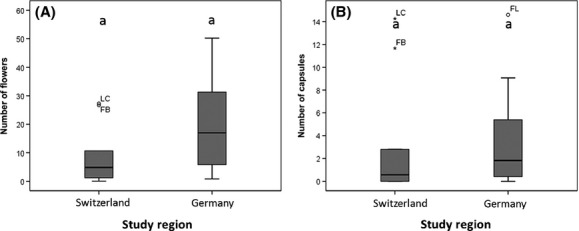
Number of flowers and capsules of *D. gratianopolitanus* of populations from Switzerland and Germany: (A) number of flowers and (B) capsules.

**Figure 4:**
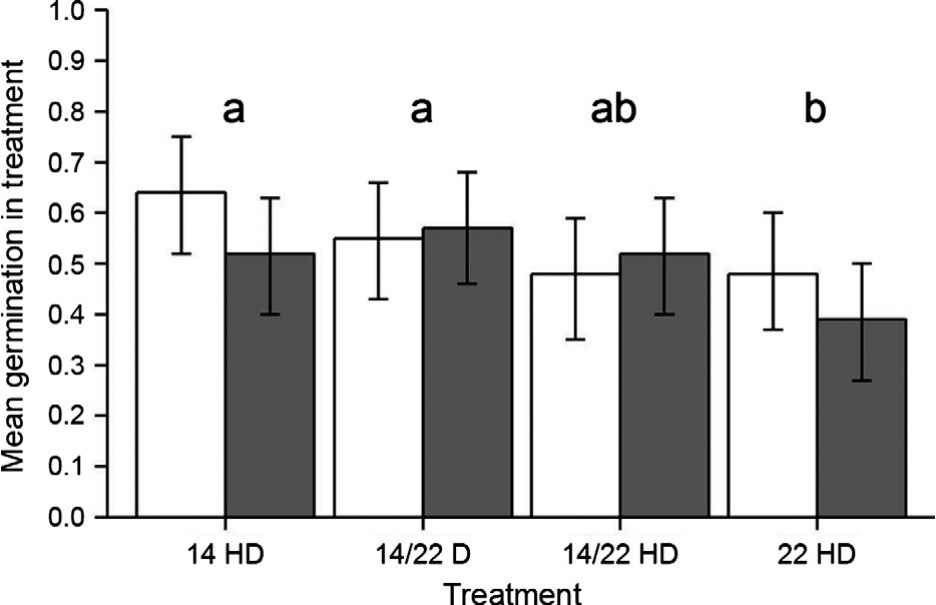
Germination of *D. gratianopolitanus* per treatment and region on day 7. Bars depict the most credible values for germination according to the Bayesian model. White bars represent germination of seeds from the Bavarian populations, while gray bars represent seed germination in Swiss populations. Error bars show lower and upper limits of 95% highest density intervals. Lowercase letters indicate credibly nonzero differences between treatments.

Our analyses revealed no credible differences in flower production (Table3) between Swiss and German populations. Moreover, the mean number of capsules developed out of flowers (3.07 vs. 3.71) was comparable in Swiss and German populations (Fig.3A,B). Therefore, regions did not differ in fruit set. Seed germination seemed to be the lowest in the 22 HD group, which differed credibly from the 14 HD and the 14/22 D groups (cf. lowercase letters in Fig.4). However, when contrasting germination between regions, no difference was apparent between Bavarian and Swiss populations (mean difference: 7%, HDI: [−0.09, 0.24]).

**Table 3:** Reproductive traits and genetic variation of all investigated populations of *D. gratianopolitanus*. Mean values for the study regions and results of the statistical analysis are given. (NF, number of flowers; NC, number of capsules; FS, fruit set; H_e_, Nei's gene diversity; I, Shannon Index; % PL, percentage of polymorphic loci)

N.	Abbr.	Population	NF	NC	FS	H_e_	I	% PL
Switzerland								
1	LC	Le Chasseron	26.67	14.27	0.54	0.141	0.217	48.38
2	CR	Col de Roches	0.07	0.00	0.00	0.093	0.144	34.09
3	CF	Chrüzflue	4.40	0.53	0.12	0.146	0.223	46.75
4	BK	Balmfluechöpfli	0.13	0.00	0.00	0.152	0.235	52.92
5	RF	Ravellen	5.40	0.60	0.11	0.130	0.200	43.18
6	BR	Bränten	1.67	0.00	0.00	0.149	0.228	49.35
7	HF	Holzflue	8.07	0.80	0.10	0.139	0.216	48.70
8	GE	Geissflue	1.20	0.00	0.00	0.154	0.236	50.65
9	FB	Ruine Froburg	27.27	11.67	0.43	0.146	0.222	46.10
10	SF	Säliflue	10.67	2.80	0.26	0.143	0.220	48.38
		Mean	8.55	3.07	0.36	0.139	0.206	46.85
Germany								
11	SL	Schwedenleite	22.80	5.40	0.24	0.156	0.234	45.78
12	FL	Fischleiten	46.67	14.60	0.31	0.153	0.233	48.05
13	RK	Rosskopf	23.67	9.07	0.38	0.141	0.211	40.91
14	KF	Kachelfels	11.20	2.80	0.25	0.167	0.249	47.40
15	FH	Falkenhorst	10.13	1.80	0.18	0.165	0.251	51.62
16	SE	Schleuse	5.80	1.20	0.21	0.152	0.233	50.65
17	SB	Steinerbrückl	5.33	1.83	0.34	0.134	0.200	38.64
18	GB	Grain am Berg	50.20	0.00	0.00	0.155	0.230	42.86
19	AC	Achtel	31.33	0.40	0.01	0.119	0.174	30.84
20	GF	Grundfelsen	0.87	0.00	0.00	0.107	0.157	27.27
		Mean	20.80	3.71	0.18	0.145	0.217	42.40
Most credible difference: HDI			12.5 [−3.18, 27.9]	0.24 [−0.99, 3.46]	0.04 [0.23, 0.11]	0.005 [−0.013, 0.024]	0.004 [−0.028, 0.031]	4.47 [−11.9, 2.51]
Effect size *r*			0.41	0.19	0.16	0.14	0.07	0.33

### Molecular analyses

**Figure 5:**
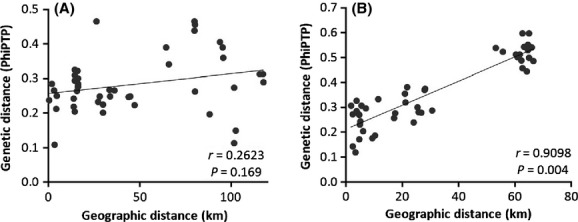
Correlation of geographic and genetic distances between populations of *D. gratianopolitanus* (Mantel test) from (A) Switzerland and (B) Germany.

AFLP analysis resulted in 308 fragments. Genetic variation within populations (Table3) was comparable in Switzerland and Germany. Percentage of polymorphic bands ranged from 34.09% to 52.92% in Swiss populations and from 27.27% to 51.62% in German populations. Mean values for polymorphic loci, Shannon's Information Index, and Nei's gene diversity were comparable between both regions. An overall analysis of molecular variance (AMOVA) revealed 14% of variation among Switzerland and Germany, 28% among populations within regions and 57% within populations (Table4) with Φ_PT_ = 0.42. In a two-level AMOVA considering all populations, we observed 38% of variation among all populations and 62% within populations. Separate two-level analyses for Switzerland and Germany resulted in a slightly higher similarity among Swiss populations (Φ_PT_ = 0.29) than among German populations (Φ_PT_ = 0.37). A mantel test revealed no significant correlation of genetic and geographic distances between populations in Switzerland (Fig.5A), but a highly significant correlation (*r* = 0.9098, *P* = 0.004) in Germany (Fig.5B).

**Table 4:** Summary of the conducted analyses of molecular variance (AMOVA) with the studied populations of *D. gratianopolitanus*. (df, degree of freedom; SS, sums of squares; MS, mean squares; %, percentage of molecular variance; Φ_PT_, genetic variation). All results were verified with *P* < 0.001

Molecular variation	df	SS	MS	%	Φ_PT_
All 20 populations grouped in two regions					
Among regions	1	1108.988	1108.988	14	0.42
Among populations within regions	18	3639.084	202.171	28	
Within populations	277	6727.767	24.288	57	
All 20 populations together					
Among populations	19	4748.072	249.899	38	0.38
Within populations	277	6727.767	24.288	62	
10 populations from Switzerland					
Among populations	9	1578.940	175.438	29	0.29
Within populations	140	3497.067	24.979	71	
10 populations from Germany					
Among populations	9	2060.144	228.905	37	0.37
Within populations	137	3230.700	23.582	63	

### Correlation analyses

Generally, we observed in all analyses (Table5) a significant correlation between habitat area and populations density as well as between the three measurements of genetic variation (Nei's gene diversity, Shannon's Information Index, and percentage of polymorphic loci). This is, however, not unexpected, as these values are based on the same raw data.

**Table 5:** Results of the Spearman correlation analyses for all studied populations and for the populations from both regions separately. Significant results are given in bold. (HA, habitat area in m²; NI, number of individuals; PD, population density in cushions per m²; CS, cushion size in cm²; CD, cushion density in shoots per m²; NF, number of flowers; NC, number of capsules; H_e_, Nei's gene diversity; I, Shannon Index; % PL, percentage of polymorphic loci)

	HA	NI	PD	CS	CD	NF	NC	He	I
20 populations from Switzerland and Germany									
NI	0.181								
PD	−0.958^*^^*^	0.010							
CS	−0.345	0.518^*^	0.461^*^						
CD	−0.544^*^	0.060	0.645^*^^*^	0.164					
NF	−0.383	0.434	0.455^*^	0.239	0.438				
NC	−0.094	0.486^*^	0.164	0.191	0.146	0.636^*^^*^			
He	−0.009	0.245	0.121	0.039	0.189	0.276	0.184		
I	0.138	0.258	−0.013	0.073	0.053	0.118	0.121	0.967^*^^*^	
%PL	0.571^*^^*^	0.395	−0.464^*^	0.109	−0.313	−0.156	0.036	0.586^*^^*^	0.738^*^^*^
10 populations from Switzerland									
NI	0.165								
PD	−0.976^*^^*^	−0.055							
CS	0.006	0.488	0.03						
CD	−0.091	−0.311	0.139	−0.818^*^^*^					
NF	−0.224	0.829^*^^*^	0.309	0.333	−0.115				
NC	−0.131	0.856^*^^*^	0.188	0.338	−0.231	0.957^*^^*^			
He	0.018	−0.294	0.012	0.377	−0.237	−0.182	−0.358		
I	0.006	−0.305	0.018	0.394	−0.273	−0.212	−0.381	0.997^*^^*^	
%PL	0.492	−0.131	−0.511	0.316	−0.292	−0.219	−0.320	0.762^*^	0.766^*^^*^
10 populations from Germany									
NI	0.569								
PD	−0.866^*^^*^	−0.214							
CS	−0.024	0.506	0.328						
CD	0.377	0.366	−0.207	0.345					
NF	−0.134	0.262	0.267	−0.018	0.442				
NC	0.345	0.180	−0.216	−0.091	−0.006	0.219			
He	0.353	0.366	−0.207	−0.406	−0.115	0.261	0.347		
I	0.524	0.502	−0.305	−0.243	−0.049	0.116	0.421	0.948^*^^*^	
%PL	0.632^*^	0.726^*^	−0.328	0.127	0.261	0.103	0.359	0.770^*^^*^	0.900^*^^*^

Level of significance indicated by asterisks (^*^^*^: *p* < 0.01, ^*^: *p* < 0.05)

Considering all populations from both study regions (Table5), we observed significant positive correlations between number of individuals and cushion size (ρ = 0.518), population density and cushion size (ρ = 0.461), as well as population density and cushion density (ρ = 0.645). Moreover, there was a positive correlation between number of flowers and population density (ρ = 0.455), number of capsules and number of individuals (ρ = 0.486), and number of flowers and number of capsules (ρ = 0.636). We also observed a negative correlation between habitat area and cushion density (ρ = −0.544). Finally, genetic variation (%PL) was positively correlated with habitat area (ρ = 0.571) and negatively with population density (ρ = −0.464). This means that cushions are larger and denser in larger and denser populations and that more flowers and capsules are produced in denser populations. Genetic variation was, however, higher in populations colonizing a larger habitat area with a lower density.

If we analyzed the two regions separately (Table5), we found less significant correlations. In Swiss populations, cushion size correlated negatively with cushion density (ρ = −0.818). Furthermore, we observed positive correlations between number of individuals and number of flowers (ρ = 0.829), number of individuals and number of capsules (ρ = 0.856), as well as between number of flowers and number of capsules (ρ = 0.957). Genetic variation within Swiss populations was, however, not correlated significantly with any of the population structure parameters or reproductive traits. This means that large cushions were less dense in Switzerland and that more flowers and capsules were produced in larger populations.

In German populations (Table5), we observed no significant correlation between population structure parameters and reproductive traits. Genetic variation (%PL) was, however, correlated significantly positive with habitat area (ρ = 0.632) and number of individuals (ρ = 0.726), which means that larger populations exhibited higher genetic variation.

## Discussion

In the study presented here, we analyzed the impact of isolation on the population structure, reproduction, and genetic variation of the rare plant species *D. gratianopolitanus*. In contrast to our assumptions, population density was higher in Germany, although the magnitude of isolation was higher in this study region. This supports the findings of a previous study on *Hornungia petraea* (Kluth and Bruelheide 2005), which also reported higher density in isolated peripheral populations. However, in a study on *Cirsium acaule* and *C. heterophyllum* (Jump and Woodward 2003), lower population density was found in more isolated populations. One reason for these differing observations may be the environmental conditions in the study regions. The magnitude of population isolation is indeed higher in Germany than in Switzerland, which supports the assumption that populations of a species become progressively less frequent and more spatially isolated approaching the edge of a species' range (Lawton 1993; Sagarin and Gaines 2002; Sagarin et al. 2006). However, environmental conditions must not necessarily get less suitable with increasing isolation. Under benign conditions, population density may increase despite increasing isolation. These conditions could also be the reason why we found higher cushion density in more isolated populations. Individual growth rate and, therefore, the number of shoots per square meter may be higher under suitable ecological conditions.

Increased population and cushion density affect growth and flowering traits (Hooftman et al. 2003) and are, for this reason, of particular interest for the reproduction of a plant species. Small and less dense populations often suffer from reduced pollinator activity (Honnay and Jacquemyn 2007) and exhibit decreased levels of fitness (Leimu et al. 2006). Increased population and cushion density may, therefore, explain why we observed no differences in reproductive traits between the two regions with a different magnitude of isolation. There was even a trend for higher flower production in the region with a higher magnitude of isolation, although it must be taken into account that we analyzed flower production only in one vegetation period. In a study on *Succisa pratensis*, similar observations were reported. In this survey, plants from more strongly isolated habitat islands also produced on average more flower heads and rosettes than plants from connected habitat islands (Hooftman et al. 2003). However, the higher number of flowers in Germany may be well explained by the higher population density in this study region.

Although we expected a negative impact of stronger isolation on the reproduction of *D. gratianopolitanus*, similar germination rates were found in both analyzed study regions. It is already known that increased habitat fragmentation and the associated isolation may cause a decline in seed yield (Steffan-Dewenter and Tscharntke 1999). For *Juniperus communis*, the production of filled seeds declined toward the limits (García et al. 2000) and strongly isolated populations of *Narthecium ossifragum* produced more, but smaller seeds that failed to germinate (Tsaliki and Diekmann 2009). However, a study on *Lychnis viscaria* revealed no difference between more and less isolated populations (Lammi et al. 1999) and in a recent study on *Draba aizoides*, strongly isolated relict populations exhibited even higher germination rates than populations from the center of the distribution range (Vogler and Reisch 2013). High population density and the broad pollinator spectrum of *D. gratianopolitanus* accompanied by a strong potential for long-distance pollination (Erhardt 1990) may guarantee an effective pollination and the production of fertile seeds with high germination rates also under conditions of stronger isolation, as reported in our study here.

The level of genetic variation within populations of *D. gratianopolitanus* was within the range observed for rare, perennial and outcrossing plant species (Reisch and Bernhardt-Römermann 2014). However, we observed no significant difference between the study regions, which means that higher isolation in Germany compared to Switzerland did not result in genetic depauperation. In a previous review, it was already demonstrated that rare species are less susceptible to the effects of fragmentation than common species (Honnay and Jacquemyn 2007), which supports the assumption that naturally isolated populations are affected to a lower extent by the negative consequences of isolation such as the loss of variation and inbreeding (Huenneke 1991). For a set of isolated alpine plant species, it has quite recently been demonstrated that natural fragmentation does not necessarily result in the loss of genetic variation (Kuss et al. 2008), which supports our results. In particular, postglacial recolonization processes can mask the impact of isolation on genetic variation, as they affect either demography or population genetic structure directly (Pfeifer et al. 2009).

In the case of *D. gratianopolitanus*, genetic variation within populations seems to be much more affected by population density and size than by isolation. We could show that genetic variation decreases with increasing population density across all studied populations. This may be traced back to the equalizing effects of gene flow within populations, which should be stronger in dense populations. In the German study region with a higher magnitude of isolation, genetic variation within populations depended furthermore on population size, which is already known as most important factor for genetic variation within populations (Leimu et al. 2006). The lack of this relationship in the Swiss study region with a lower magnitude of isolation may be attributed to the gene flow between populations, which presumably buffers the effect of population size and generates higher levels of genetic variation also within smaller populations.

Genetic variation was higher between populations of *D. gratianopolitanus* in Germany than in Switzerland. Moreover, a correlation of genetic and geographic distances occurred only between German but not between Swiss populations. It has been postulated that genetic drift is 2–30 times higher in isolated peripheral populations (Vucetich and Waite 2003) and it is beyond all question that gene flow counteracts the effects of genetic drift (Slatkin 1987). The stronger isolation of German populations apparently restricts gene flow and seems to cause a more distinct pattern of geographic variation in this study region.

Based upon our results, it can be concluded that the isolation of naturally fragmented populations must not necessarily have a negative impact on population structure, reproduction and genetic variation within populations. However, genetic variation between populations increases with geographic isolation due to restricted gene flow. Isolated populations make, from this point of view, due to their genetic uniqueness an essential contribution to a species' full evolutionary potential. The protection of isolated *D. gratianopolitanus* populations should, therefore, be an integral part of the strategy to conserve this central European endemic plant species.
